# Genetic determinants of cocaine-associated agranulocytosis

**DOI:** 10.1186/s13104-015-1219-4

**Published:** 2015-06-13

**Authors:** Jane A Buxton, John Omura, Margot Kuo, Colin Ross, Despina Tzemis, Roy Purssell, Jennifer Gardy, Bruce Carleton

**Affiliations:** BC Center for Disease Control, 655 West 12th Avenue, Vancouver, BC V5Z 4R4 Canada; School of Population and Public Health, University of British Columbia, 2206 East Mall, Vancouver, BC V6T 1Z3 Canada; Child and Family Research Institute, 950 West 28th Avenue, Vancouver, BC V5Z 4H4 Canada; Drug and Poison Information Centre, BC Centre for Disease Control, 655 West 12th Avenue, Vancouver, BC V5Z 4R4 Canada

**Keywords:** Agranulocytosis, Levamisole, Cocaine, HLA-B27 antigen, Pharmacogenetics, Neutropenia

## Abstract

**Background:**

Drug-induced agranulocytosis is a recognized adverse drug event associated with serious infectious complications. Levamisole is an antihelminthic and immunomodulator withdrawn from North American markets in 2005 after reports of agranulocytosis. Previous studies of levamisole, suggest that the human leukocyte antigen (HLA)-B27 confers a genetic predisposition to this adverse drug event. Since 2009, emergency room visits due to agranulocytosis in individuals consuming levamisole-adulterated crack-cocaine have increased.

**Methods:**

We performed a case–control study using a genotyping assay and novel gene chip to test the association between cocaine-associated agranulocytosis (CAA) and HLA-B27 and to identify pharmacokinetic and pharmacodynamic gene variants associated with the phenotype.

**Results:**

Fifty-one CAA cases were identified through a provincial physician reporting system between 2008 and 2011. We examined eight of these cases and 26 matched controls. Genotyping revealed a significant association between HLA-B27 and CAA (odds ratio [OR] 9.2, 95% confidence interval [CI], 1.54–54.6). We also observed a similar association with the HLA-B27 tag single nucleotide polymorphism rs4349859 (OR 9.2, 95% CI 1.5–54.6) and rs13202464 (OR 6.7, 95% CI 1.1–41). Further associations were identified with variants in the *ARBCC12* (OR 10.0, 95% CI 2.7–36.8) and *CYP11A1* (OR 7.4, 95% CI 2.1–26.6) genes, while a novel protective association was observed with variants in the *ARDB1* gene (OR 0.06, 95% CI 0.007–0.46).

**Conclusions:**

We confirmed the association of HLA-B27 with CAA and identified additional susceptibility variants. Health care providers should inform people who are identified as having CAA that it is genetically determined and can recur with continued cocaine use. The severity of infections and subsequent hospitalization, and the risk of recurrence may prompt health-promoting behaviour changes of the affected individuals. These genetic associations warrant the attention of public health and knowledge translation efforts to highlight the implications for susceptibility to this severe adverse drug event.

## Background

Agranulocytosis is a recognized adverse drug event characterized by a decrease in peripheral neutrophil count to less than 500/µL (0.5 × 10^9^/L) [1], with affected individuals at increased risk of infection, sepsis, and death [2]. Multiple drugs have been implicated as potential triggers of agranulocytosis, including levamisole [3]. Originally formulated as a veterinary antihelminthic, levamisole was later used to treat autoimmune disorders including rheumatoid arthritis (RA) and as adjuvant chemotherapy for colon cancer. Agranulocytosis became a well-recognized complication of levamisole therapy, occurring in 2.5–13% of exposed patients [4]. These findings eventually led to its voluntary withdrawal from the US market in 1999 [5] and the Canadian market in 2005 [6], although levamisole continues to be used in veterinary medicine as an antihelminthic in the USA, Canada and South America [5, 6].

In 2005, levamisole was first identified as a cocaine adulterant [7] and by July 2009 was found in as many as 69% of seized cocaine samples tested by the United States Drug Enforcement Administration [8]. Reports of agranulocytosis in individuals using levamisole-adulterated cocaine first emerged in 2009 from Alberta and British Columbia (BC), Canada [6, 9] and New Mexico, USA [8] with later reports from other parts of Canada and the United States demonstrating a spectrum of pathology including cutaneous vasculopathy [10–13]. Relapse is common with approximately half of patients experiencing more than one episode of agranulocytosis associated with return to cocaine use [6].

The mechanism by which levamisole induces agranulocytosis is thought to be immune-mediated. While no reported cases of levamisole-induced agranulocytosis could be identified from the period when the drug was used primarily as an antihelminthic agent, case reports emerged when its treatment profile broadened to include RA in the late 1970s and early 1980s [14, 15]. Studies of affected RA patients demonstrated an association between human leukocyte antigen (HLA)-B27 positivity and levamisole toxicity [16–18]. Initial findings amongst cases of cocaine-associated agranulocytosis (CAA) suggest a similar association with HLA-B27 [2, 15].

We performed a case–control study to confirm the association between CAA and HLA-B27 and used a novel SNP genotyping assay to identify genetic markers for CAA.

## Methods

### Setting and study design

We conducted a case–control study of CAA using the British Columbia Centre for Disease Control’s (BCCDC) voluntary physician reporting system that communicated with emergency department staff and other health professionals through provincial medical health officers. Approval was obtained from the University of British Columbia Clinical Research Ethics Board.

### Study participants

Cocaine-associated agranulocytosis cases, defined as individuals with a neutrophil count ≤0.5 per 10^9^/L and laboratory confirmation or self-reported cocaine use at the time of diagnosis, were identified both retrospectively from 2008 and prospectively from case reports provided between January 2009 and October 2011. Case reports included patient demographics, clinical information, and past medical history [6, 19]. Eligible cases were recruited through study invitation letters distributed by the reporting physician with an invitation to contact a research team member for enrollment. This study employed a 1:3 case–control ratio; controls were recruited using case-based snowball sampling (i.e. cases gave study information to people they knew to invite them to contact the research team if they were interested in participating in the study) and through a local harm reduction center. Controls were defined as individuals with no known history of agranulocytosis and self-reported current cocaine use and matched on age (±5 years), sex and ethnicity including self-reported Aboriginal and Non-Aboriginal heritage. All participants were paid $25 to complete a survey questionnaire and provide a saliva sample for genetic analysis.

### Genotyping

Genomic DNA was purified from saliva samples using the QIAmp DNA purification system (Qiagen, Toronto, ON, Canada) according to the manufacturer’s protocol. Samples were genotyped using a previously described TaqMan-based assay specific for multiple variants of the HLA-B27 haplotype, which identifies the common HLA-B27 subtypes including HLA-B2702, B2705, B2706, B2707, and B2708 [20], followed by examination of two HLA-B27 tag SNPs (rs4349859 and rs13202464) using a multiplexed SNP genotyping assay with a custom Illumina iSelect genotyping panel (Illumina, San Diego, CA, USA). This custom panel tested 7,600 genetic variations in 1,350 selected candidate genes involved in drug absorption, distribution, metabolism, and excretion (ADME) and also includes drug transporter and receptor variants. SNP genotype data were clustered manually using GenomeStudio software (Illumina, San Diego, CA, USA). The average sample call rate was 99.88% and all samples had call rates >99.6%. All SNPs described were in Hardy–Weinberg equilibrium.

### Statistical analyses

Baseline characteristics were compared using the Fisher’s exact test for categorical variables and the unpaired t test for continuous variables. Genetic results were compared using the Fisher exact test for the dominant genetic model or the exact form of the Cochran–Armitage trend test for the additive genetic model.

## Results

### Patient characteristics

As of October 2011, 51 individuals with one or more episodes of CAA were reported through the BCCDC voluntary physician reporting system. The average age was 40 years (range 22–64) and 55% were female. Ancestry information was self-reported; 51% reported Aboriginal ancestry, 33% non-Aboriginal, 4% other and 12% unknown or missing. When asked about route of cocaine administration, 45% smoked only, 20% snorted only, 2% injected only, 8% used more than one route and 24% had missing information.

Of the 51 eligible CAA subjects, 37 did not participate (predominantly due to inability to contact), 14 were enrolled, 6 were lost to follow-up (defined as initiated contact but either did not complete the questionnaire or did not provide a sample or both), and eight ultimately participated. The self-reported ancestry of the cases was predominantly Aboriginal (five); two cases identified as Caucasian and one as South Asian. Twenty-six controls were recruited and were similar to cases in terms of age, gender, ancestry (all eight non-Aboriginal were Caucasian), HIV and hepatitis C status and rheumatologic history (Table 1).

**Table 1:** Baseline characteristics of cases and controls

Characteristic	Cases (N = 8)	Controls (N = 26)	P value
Sex, no. (%)			0.681
Female	6 (75)	16 (62)	
Male	2 (25)	10 (38)	
Mean age, year (range)	37 (27–48)	39 (28–53)	0.617
Self reported ancestry, no. (%)			
Aboriginal	5 (62)	18 (69)	
Non-aboriginal^a^	3 (38)	8 (31)	
HIV positivity, no. (%)	1 (14)	4 (15)	1
Hepatitis C positivity, no. (%)	1 (14)	12 (46)	0.116
History of rheumatoid arthritis, no. (%)	1 (14)	2 (8)	1
History of unusual skin problems, no. (%)	1 (14)	3 (12)	1

^a^Non aboriginal: cases 2 Caucasian, 1 South Asian; Controls all Caucasian.

Although not systematically asked, two of the cases reported stopping cocaine use following their hospitalization for CAA.

### Association of CAA with HLA-B27

Taqman-based genotyping of the HLA-B27 haplotype revealed a significant association with CAA (Fisher exact p value = 0.017, odds ratio (OR) = 9.2, 95% confidence interval [CI], 1.5–54.6) (Table 2), replicating the previously identified association of HLA-B27 with CAA. Overall, 62.5% of CAA cases carried the HLA-B27 allele, compared to 15.4% of the controls.

**Table 2:** HLA-B27 variants associated with cocaine-associated agranulocytosis

SNP haplotype	Cases (N = 8)	Controls (N = 26)	Odds ratio (95% CI)	P value
HLA-B27			9.2 (1.54–54.6)	0.017
+, no. (%)	5 (62.5)	4 (15.4)		
−, no. (%)	3 (37.5)	22 (84.6)		
rs4349859—dominant model			9.2 (1.54–54.6)	0.017
A_, no. (%)	5 (62.5)	4 (15.4)		
GG, no. (%)	3 (37.5)	22 (84.6)		
rs4349859—additive genetic model				0.017
AA, no. (%)	1 (12.5)	0		
AG, no. (%)	4 (50)	4 (15.4)		
GG, no. (%)	3 (37.5)	22 (84.6)		
rs13202464—dominant model			6.8 (1.11–41.0)	0.042
G_, no. (%)	6 (75)	8 (30.8)		
AA, no. (%)	2 (25)	18 (69.2)		
rs13202464—additive genetic model				0.011
GG, no. (%)	2 (25)	0		
AG, no. (%)	4 (50)	8 (30.8)		
AA, no. (%)	2 (25)	18 (69.2)		

*SNP* single nucleotide polymorphism, *CI* confidence intervals.

Using a multiplexed SNP genotyping assay, an identical association in complete linkage disequilibrium was observed between CAA and a 41 kb away HLA-B27 tag SNP rs4349859 (Fisher exact p value = 0.017, OR = 9.2 (95% CI 1.5–54.6) under a dominant genetic model and an additive genetic model (Fished exact p value 0.017) (Table 2). For a retrospective prediction of CAA, the TaqMan HLA-B27 test and rs4349859 SNP exhibited a sensitivity of 62.5% and specificity of 84.6%, and a positive predictive value (PPV) of 71.4% and negative predictive value (NPV) of 88.0%.

A second tag SNP, rs13202464, located 20 kb away and in LD with the tested HLA-B27 haplotype (Our cohort r^2^ = 0.55) and 21 kb away and in LD with rs4349859 (Our cohort r^2^ = 0.47; HapMap CEU D’ 1.0, r^2^ = 0.32) showed a significant, though slightly weaker association (Fisher exact p value = 0.042, OR = 6.8, 95% CI 1.1–41). Compared to the TaqMan HLA-B27 haplotype test and rs4349859, the rs13202464 variant exhibited an increased sensitivity (75.0%) with a reduced specificity (69.2%), reduced PPV (60.0%) and increased NPV (90.0%).

### Novel genetic variants associated with CAA

In the hypothesis-generating chip-based analysis of the 7,600 variants in the pharmacokinetic and pharmacodynamics SNP panel, a significant association was identified for a protective intergenic SNP near the *ADRB1* (adrenoceptor beta 1) gene (exact Armitage p value = 9.1 × 10(−6); p value = 0.046 after Bonferroni correction for multiple testing; estimated allelic odds ratio = 0.06 (95% CI 0.007–0.46) (Table 3). In addition, two other genetic variants exhibited strong trends (P value <0.001) but were not significantly associated after multiple testing correction in the *ABCC12* (ATP binding cassette subfamily C member 12, OR = 10.0, 95% CI 2.7–36.8) and *CYP11A1* (cytochrome P450 family 11 subfamily A polypetide 1, OR = 7.4, 95% CI 2.1–26.6) genes (Table 3). The variant in *ABCC12* is a non-synonymous variant causing an Arg1117Cys amino acid change carried by 35% of the patients in this study, with relatively similar frequencies worldwide except for a higher frequency in some African populations. The intronic *CYP11A1* variant was present in 75.0% of CAA cases, compared to 28.8% of controls. This variant exhibits relatively similar allele frequencies worldwide (27–49%).

**Table 3:** Additional genetic variants associated with cocaine-associated agranulocytosis

SNP	Gene	Chr	Alleles	Type	Allele frequency (%)				
					Cases	Controls	Odds ratio (95% CI)	P value	Corrected P value
rs10885531	*ADRB1*	10	G/A	Intragenic	0	53.8	0.06 (0.007–0.46)	9.09 × 10^−6^	0.043
rs7193955	*ABCC12*	16	A/G	Nonsynonymous	75	23.1	10 (2.7–36.8)	1.00 × 10^−4^	0.47
rs2279357	*CYP11A1*	15	G/A	Intronic	75	28.8	7.4 (2.1–26.6)	7.29 × 10^−4^	1

*SNP* single nucleotide polymorphism, *CI* confidence intervals, *Chr* chromosome.

## Discussion

Agranulocytosis is a severe adverse drug event emerging as an important complication of crack cocaine use in Canada. Here, we report the first case–control study of the pharmacogenomic determinants of CAA. We demonstrate a significant replication of the association between CAA and HLA-B27 using both the standard TaqMan assay for HLA-B27 and two tag SNP markers previously associated with HLA-B27. A novel protective association was identified between CAA and an intergenic variant near the *ADRB1* gene, with adverse associations identified between CAA and variants in the *ABCC12* and *CYP11A1* genes. Our results confirm the genetic basis for the association between levamisole and CAA, a finding with important implications for identifying and managing at-risk individuals to prevent occurrence and relapse.

HLA-B27 has been implicated in a wide range of pathology, including spondyloarthropathies; however, the genetic mechanism by which HLA-B27 acts in the autoimmune disease pathway remains largely undetermined [21]. One hypothesis, known as the linked gene theory, suggests that HLA-B27 is a marker for a nearby true susceptibility locus [22], while other studies suggest it is not the sole mediator of pathology and its effects are modulated by other HLA and non-HLA genes [23]. Indeed, we observe a novel protective association between CAA and an intergenic variant near the *ADRB1* gene, with adverse associations identified between CAA and variants in the *ABCC12* and *CYP11A1* genes. *ADRB1* encodes the beta-1 adrenergic receptor, which when bound by catecholamines stimulates a sympathetic nervous system response important in the regulation of cardiac function. Although levamisole contains neither a catechol nor an amine group, a single report in the literature has linked levamisole to ADRB1 activity. Ogunbiyi et al. reported that in a bovine model, levamisole restored ADRB1 function—as measured by the productive of reactive oxygen species—in pulmonary alveolar macrophages in response to infection with two distinct respiratory viruses [24]. Given the protective effect of the *ADRB1* variant reported in the present study and this previous work reporting a beneficial immunomodulatory effect of levamisole in a phagocytic cell population, this association between *ADRB1* and CAA merits further investigation.

Although the case–control study design supports the presence of an association, the sample population was small. Challenges were encountered in recruiting both cases and controls. As cases were identified through a voluntary physician reporting system, the busy clinical environment may have impeded enrollment. People with problematic substance use may discharge themselves against medical advice, before they were approached to participate in the study [25]. Moreover the target population was highly transient, with homelessness and lack of contact information following hospital discharge hampering follow-up efforts. Recruitment of controls was first attempted using case-based snowballing sampling, which ultimately produced few participants; therefore active recruitment was performed in a high-risk neighborhood. These issues are important considerations for future research in this vulnerable and at-risk population, particularly in situations where a timely response is warranted. Furthermore, our case definition included both confirmed and probable cases, as we were unable to confirm the presence of levamisole in probable cases. It was assumed that persons who habitually used cocaine would likely be exposed to levamisole-tainted cocaine at some point during the study period due to the high prevalence of this adulterant in circulating crack-cocaine [8]. However, since controls were also persons using crack-cocaine with undetermined levamisole presence, this exposure misclassification would be true for both groups and likely bias towards the null. Replication of the results using a larger sample and examining a dose–response relationship would further contribute to our understanding of CAA.

Levamisole-induced agranulocytosis is an emerging clinical and public health concern among individuals using crack cocaine, and the potential complications of this adverse drug event are important considerations in the clinical care of this vulnerable population. A deeper understanding of CAA has allowed cases to be diagnosed and managed without invasive procedures such as a bone biopsy [6] and has facilitated efforts to inform people who use drugs about the importance of seeking medical assistance promptly when symptoms occur. In patients presenting with unexplained fever and agranulocytosis, clinicians are encouraged to consider the possibility of cocaine use. Recommended management of patients with CAA presenting with fever or infection includes supportive care, and treatment with empiric intravenous broad spectrum antibiotics and granulocyte-colony stimulating factor (G-CSF or filgrastim) [6, 9].

## Conclusion

Although our sample size was small the strength of the association confirms the primary hypothesis of a genetic association between CAA and HLA-B27. It is not practical to test people who use cocaine for HLA-B27 status; however health care providers should warn people who are identified as having that that it is genetically determined they are at high risk of reoccurrence if they continue to use cocaine. As described by the health belief model, the perceived susceptibility and risk of recurrence of CAA, and the severity of the outcome and hospitalization may act as an internal cue to prompt health-promoting behavior such as stopping cocaine use or engaging in addiction treatment [26]. These genetic associations warrant the attention of public health and knowledge translation efforts to highlight the implications for susceptibility to this severe adverse drug event that may occur repeatedly due to chronic cocaine use.
